# Management of Extranodal Marginal Zone Lymphoma: Present and Upcoming Perspectives

**DOI:** 10.3390/cancers14123019

**Published:** 2022-06-19

**Authors:** Dominic Kaddu-Mulindwa, Lorenz Thurner, Konstantinos Christofyllakis, Moritz Bewarder, Igor Age Kos

**Affiliations:** Department of Internal Medicine I, Hematology and Oncology, Rheumatology and Clinical Immunology, Saarland University Medical Center, 66424 Homburg, Germany; lorenz.thurner@uks.eu (L.T.); konstantinos.christofyllakis@uks.eu (K.C.); moritz.bewarder@uks.eu (M.B.); igor.kos@uks.eu (I.A.K.)

**Keywords:** marginal zone cell lymphoma, MALT, management, indolent lymphoma

## Abstract

**Simple Summary:**

Extranodal marginal zone lymphoma distinguishes itself from other indolent lymphomas due to its unique pathophysiology and natural history. This is reflected in its management, where next to traditional treatment strategies such as observation, radiotherapy or chemotherapy, eradication of the causal agent and even surgery represent important aspects of therapy. This review focuses on the particular aspects of this indolent lymphoma that affect management and summarizes the current evidence and different guidelines.

**Abstract:**

Extranodal marginal zone lymphoma (EMZL) encompasses a subgroup of non-Hodgkin lymphomas that often present with localized involvement and may manifest in a diversity of organs and tissues. EMZL pathogenesis is in some cases linked to chronic inflammation/infection, which may impose additional diagnostic and clinical challenges. The most studied and established connection is the presence of *Helicobacter pylori* in gastric EMZL. Due to its heterogeneity of presentation and intricate pathological features, treatment can be complex, and staging systems are decisive for the choice of therapy. Nevertheless, there is no consensus regarding the most suitable staging system, and recommendations vary among different countries. As a rule of thumb, in limited stages, a local therapy with surgery or radiation is the preferred option, and it is potentially curative. Of note, eradicating the causal agent may be an important step of treatment, especially in gastric EMZL, in which Helicobacter pylori eradication remains the first-line therapy for the majority of patients. In patients with more advanced stages, watch-and-wait is a valuable option, especially amongst those without clear indications for systemic therapy, and it may be carried on for several years. If watch-and-wait is not an option, systemic therapy may be needed. Even though several agents have been tested as monotherapy or in combination in recent years, there is no consensus regarding the first-line therapy, and decisions can vary depending on individual factors, such as age, clinical performance and stage. This review aims to discuss the several aspects of EMZL, including genetic milieu, pathogenesis and staging systems, that may influence the choice of therapy. In addition, we present a summary of evidence of several systemic therapies, compare different recommendations worldwide and discuss future perspectives and novelties in its therapy.

## 1. Introduction

Marginal zone lymphoma (MZL) is a subgroup of indolent B-cell non-Hodgkin lymphomas (NHL), which share common histologic and immunophenotypic features [1]. MZL originate and/or have a stage of differentiation of B-lymphocytes in the marginal zone of secondary lymphoid follicles and commonly expresses typical markers such as CD19, CD20 and CD22, usually lacking the expression of CD5, CD10, CD23 and Cyclin D1, differentiating it from chronic lymphatic leukemia (CLL) and most germinal-center and mantle-zone derived lymphomas [2,3]. In addition, MZL may present with monoclonal gammopathy [4]. Regarding the immunophenotype, hairy cell leukemia can be a differential diagnosis as it usually shows a similar expression pattern [5]. Even though common phenotypic and genotypic characteristics are seen across all subtypes of MZL, its clinical presentation is heterogeneous, and it is therefore further classified into the following subtypes: extranodal marginal zone lymphoma (EMZL), nodal marginal zone lymphoma (NMZL), primary cutaneous marginal zone lymphoma (PCMZL) and splenic marginal zone lymphoma (SMZL) [6,7]. These entities differ significantly regarding their clinical and prognostic characteristics. As an example, lymph node enlargement is the most common feature of NMZL; however, it occurs less frequently in SMZL and EMZL [8,9]. SMZL is usually restrained to the spleen, but peripheral blood involvement is common, and bone marrow involvement is also observed, whereas EMZL can involve several organs, especially the stomach, which is the most frequently involved site. [10]. Therefore, treatment can vary largely amongst subtypes [11]. MZL normally presents with an indolent course and generally has a good prognosis compared to aggressive lymphomas. Its five-year overall survival at diagnosis is approximately 80% but can reach up to 95% depending on age group [12]. In contrast, aggressive B-cell NHLs such as DLBCL have a worse prognosis with a 5-year survival rate of around 60% [13]. However, secondary transformation to DLBCL is associated with a poor outcome and inferior overall survival (OS) even though it only occurs with an annual incidence of approximately 1% per year [14].

MZL is the second most common type of indolent B-cell NHL, accounting for 5–15% of all NHLs [9,15]. EMZL is the most common subtype of MZL, corresponding to up to two-thirds of all cases of MZL and around 7% of all new diagnosed lymphomas, with an incidence of 18.3 cases per one million person-years in the United States [15,16]. In addition, EMZL is responsible for 5% of all primary gastric neoplasms [17], explaining why, in addition to hematologists, gastroenterologists are also often confronted with this diagnosis. The focus of this review is to discuss the principles of EMZL management and other distinct clinical and pathological features that might be relevant to the decision-making process. 

### 1.1. Pathogenesis of EMZL and Its Diagnostic and Therapeutic Implications

EMZL is also described as low-grade B-cell lymphoma of mucosa-associated lymphoid tissue (MALT lymphoma) [1]. Even though the stomach is the most common site of presentation, it can be found in several organs and tissues such as ocular adnexa, salivary glands and skin [18]. This higher incidence of gastric involvement does not seem to be incidental. There is a strong correlation between MALT lymphoma and *Helicobacter pylori* (*H. pylori*) infection, highlighting an important role of chronic inflammation and antigen triggering of the B-cell receptor (BCR) in the pathogenesis of EMZL [19]. After understanding this striking correlation, it was speculated that the B-cell receptor may recognize a specific *H. pylori* antigen, thus triggering a chronic immune reaction, which over time gives rise to malignant transformation. However, specific antigens have not been identified and some evidence shows that the immunoglobulins expressed by gastric MALT lymphoma B-cells are polyreactive [20]. Craig and colleagues showed that a majority of MALT lymphoma in patients are monoclonal, but according to them, the tumor immunoglobulin heavy chain genes have undergone somatic hypermutation, and approximately half of all of their analyzed tumors showed evidence of intraclonal variation and positive and/or negative selective pressure. They found that recombinantly expressed MALT lymphoma antibodies bind with intermediate affinity to various unrelated self- and foreign antigens, including Helicobacter sonicate, immunoglobulin G (IgG), DNA, and stomach extract [20]. At an early disease stage, MALT lymphomas are considered indolent tumors with low proliferation rates and minimal risk of progression. Hence, after an undetermined period of indolent growth, MALT lymphomas can acquire genetic alterations (e.g., chromosomal translocations) and progress into more aggressive lymphomas. Of note, until now, it is still not fully understood if these genetic alterations occur over time or if they are already present at lymphomagenesis [19].

Considering the inflammatory background of gastric MALT lymphomas, the eradication of *H. pylori* has been established as a fundamental part of therapy in gastric EMZL [21]. Therefore, the diagnosis of a coexisting *H. pylori* infection plays an important role in the management of MALT lymphoma. Current guidelines recommend performing serology, a urea breath test and/or a stool antigen test for ruling out an infection, even if there is no proof of infection in the immunohistology examination of mucosa biopsies. Some MALT lymphoma patients are still responsive to eradication therapy, even in the case of *H. pylori*-negative disease [22]. One possible explanation for this phenomenon may come from the presence of *Helicobacter* species other than *H. pylori* (*non*-*Helicobacter pylori Helicobacters*, NHPHs). For example, *Helicobacter heilmannii s.s* has been found in patients with *H. pylori*-negative MALT lymphoma, and in animal models, it has also been shown to cause MALT lymphoma [23,24]. Other species with the potential to trigger MALT lymphoma are *Helicobacter suis*, *Helicobacter felis*, *Helicobacter suis*, *Helicobacter bizzozeronii* and *Helicobacter salomnois* [25,26,27,28,29]. Interestingly, *H. pylori* infection has been shown to activate the p38 MAPK pathway, upregulating PD-1 expression by gastric epithelial cells and reducing immune response [30]. In addition, CagA, a cytotoxin-associated gene A expressed by *H. pylori*, can activate extracellular signal-regulated kinase (ERK) and p38 mitogen-activated protein kinase (MAPK) and upregulate BCL-2 and BCL-xL, leading to the promotion of proliferation and the inhibition of apoptosis of B-lymphocytes [31].

In addition to *H. pylori* infection, several other diseases that are accompanied by chronic inflammation have also been correlated with the occurrence of MALT lymphoma [32]. Patients with autoimmune diseases such as Sjögren’s syndrome [33], systemic lupus erythematosus (SLE), rheumatoid arthritis (RA) [34], and Hashimoto’s thyroiditis [35]. have been reported to be at higher risk of developing EMZL with or without coexisting infections. In detail, Sjögren syndrome is associated with a 6.6-fold increased risk of NHL overall, a 30-fold increase in the risk of MZL, and a 1000-fold increased risk of parotid gland EMZL [36]. Moreover, SLE is associated with a 2.7-fold increase in the risk of NHL overall and a 7.5-fold increased risk of MZL [37,38]. It is assumed that immunogenic endogenous antigens (auto-antigens) could provide a permanent growth signal for B-cells expressing appropriate BCRs, ultimately leading to increased proliferation with the accumulation of genetic alterations [39]. Moreover, there are other microorganisms associated with the occurrence of EMZL, e.g., *Chlamydia psittaci* [40], for ocular adnexa EMZL or *Campylobacter jejuni* [41] (*C. jejuni*) for small-intestine MZLs (e.g., immunoproliferative small intestinal disease, IPSID, a form of MALT lymphoma). *Achromobacter xylosoxidans* is a Gram-negative bacterium with low virulence whose influence in EMZL-lymphomagenesis remains investigational [42]. Considering its inflammatory background, diagnosing EMZL can be challenging, both from a clinical and pathological point of view, as it can be masked by these concomitant diseases. In the histological workup, surrounding inflammation may be misleading and reactive lesions are a common differential diagnosis [43]. Nevertheless, the evidence of immunoglobulin light chain restriction or clonal IgH rearrangements is usually not found in reactive processes and may be an important diagnostic tool to differentiate these entities [44]. 

As described above, MALT lymphoma is considered to be an antigen-dependent disease, even though no specific antigens have been yet identified. On a molecular level, prolonged antigen presentation results in binding of B-cell leukemia/lymphoma-10 protein (BCL10) to MALT lymphoma-associated translocation-1(MALT1) protein, resulting in activation of the nuclear factor kappa B (NF-kB) pathway promoting B-cell survival [45,46]. In *H. pylori*-negative patients, mutations in NF-κB signaling pathways may be seen in up to 40% of cases, exemplarily TNFAIP3 = 23%, CARD11 = 9%, MAP3K14 = 9% [30]. Even though this is an antigen-dependent process, genetic alterations leading to increased pathway activity may overcome the necessity for antigen stimulation, playing a pivotal role in later MALT lymphomagenesis [47]. Of note, it is not clear at which time point of lymphomagenesis the translocations are acquired. Important chromosomal aberrations involved in the pathogenesis of EMZL are translocations involving MALT1 t(11;18)(q21;q21), t(14;18)(q32;q21) or t(1;14)(p22;q32) and t(3;14)(p13;q32) and trisomy 3 [48]. 

Of special clinical interest is the t(11;18)(q21;q21), which has been correlated with poor response to *H. pylori* eradication, even in patients with early-stage disease [49]. In addition, patients with this translocation are mostly *H. pylori*-negative, show a more advanced stage at presentation but at the same time have a lower risk of secondary transformation into diffuse large B cell lymphoma (DLBCL) [50]. Regarding treatment response in this subgroup of patients, a retrospective study with 17 patients showed no influence of these translocations on treatment response to cladribine [51]. Similarly, a phase II study that investigated rituximab and bendamustine as combination therapy, which also included patients with t(11;18), showed no significant differences in treatment response with respect to the presence of this translocation [52]. However, t(11;18) has been correlated with resistance to oral alkylating agents in patients with gastric MALT [53]. Nevertheless, the influence of this mutation on therapy still needs to be prospectively evaluated in larger cohorts. 

Forkhead box protein P1 (FOXP1) is a protein located at 3p13 and is responsible for the regulation of the expression of proteins of the FOX family, such as Rag1 and Rag2, which play an important role in the development of B lymphocytes [54]. In detail, t(3;14)(p13;q32) rearranges the FOXP1 gene closer to the IgH gene resulting in an increased expression of the protein. Overexpression of FOXP1 has been correlated with poor clinical outcome in MALT lymphomas and higher risk of secondary transformation into DLBCL [48].

### 1.2. Clinical Presentation

EMZL may be found as malignant transformed lymphatic tissue in a variety of epithelial tissues, and therefore, clinical presentation is heterogeneous and depends on involved sites. The stomach is the most often affected organ, accounting for approximately 35% of all cases [15]. As mentioned above, this anatomic site is closely related to *H. pylori* infection, and therefore, causal therapy of *H. pylori* plays an important role in the management of patients with gastric MALT lymphoma. In addition, detection of t(11;18) may provide additional information regarding the possible response to antibiotic therapy [49]. 

Even though it is less common, MALT lymphoma may be found in other parts of the gastrointestinal tract, such as the small intestine, which has been correlated with *C. jejuni* infection [41,55]. In addition, *C. jejuni* has also been correlated with IPSID (also known as alpha chain disease or Mediterranean lymphoma), which is a variant of EMZL that primarily occurs in young adults in the Middle East, North and South Africa, and the Far East. Most patients ultimately relapse with an aggressive B-cell lymphoma. For such patients, treatment is similar to the management of histological transformation of follicular lymphoma into DLBCL, meaning the patient should be treated according to the more aggressive histology as it determines the outcome [56]. 

Another commonly involved site is the ocular adnexa accounting for approximately 13% of cases. Involvement of this site is often correlated with *C. psittaci* infection [40]. Similar to other MALT lymphomas, it is recommended to look for *C. psittaci* infection in biopsy samples, even though there might be great geographical variation since a correlation between MALT lymphomas of the ocular adnexa and *C. psittaci* infection could not be found in some countries [11,40,49,57,58]. Further anatomical sites that may be affected by MALT lymphoma are the salivary glands (around 8% of cases), which is commonly associated with Sjogren syndrome [59], and the skin (around 9% of cases), which can be associated with *Borrelia burgdorferi* (*B. burgdoferi*) infection. Histopathologic work-up of skin biopsies showing MALT lymphoma should therefore include a search for *B. burgdorferi* infection as recommended by current guidelines [11,60,61]. Of note, there is a case report raising the question as to whether cutaneous MALT might be associated with *Borrelia afzelii* [62]. Lungs (around 9% of cases), the thyroid gland (around 2% of cases), and the mammae (3% of cases) may also be primary sites of MALT lymphoma involvement [1]. 

Even though the general concepts of therapy are similar for different anatomical sites, therapy might differ slightly due to the heterogeneity of presentation and variety of possible causal agents. Site-specific therapeutic details will be further discussed below. 

### 1.3. Diagnosis, Staging and Prognostic Scores

A biopsy remains the mainstay for the diagnosis of EMZL, which follows the WHO classification [1]. Current guidelines recommend an immunohistochemistry panel including at least CD20, CD10, CD5, CD23, cyclin D1 and IgD with diagnostic evaluation by a specially trained hematopathologist [11]. As mentioned above, if there is diagnostic uncertainty, re-biopsy is indicated to rule out reactive lesions. In the case of gastric EMZL, biopsy material needs to be investigated for the presence of *H. pylori* infection. In analogy to this, infection with *C. jejuni, B. burgdorferi* and *C. psittaci* should be excluded in MALT lymphoma biopsies of the small intestine, the skin and the ocular adnexa, respectively. Hepatitis B virus is not commonly involved in the pathogenesis of EMZL; however, due to the risk of viral reactivation during treatment, assessment of HBV serology is also indicated [11]. Even though the hepatitis C virus is most commonly associated with SMZL, performing serological testing for it at the diagnosis of EMZL may also be helpful [63,64]. Important differential diagnoses of EMZL include reactive lesions, nodal and splenic marginal zone and other B-cell lymphomas.

EMZL mostly presents with an indolent clinical course, and taking into account its heterogeneity regarding clinical course, sufficient initial staging is crucial to guide treatment, as local therapy or a watch-and-wait strategy are feasible approaches in many cases. Retrospective data from patients with phase IE primary pulmonary MALT lymphoma showed no differences in OS between patients managed with watch-and-wait and timely immunotherapy or immunochemotherapy [65]. Moreover, watch-and-wait seems to be a safe strategy in patients with minimal histological residuals or even persisting endoscopic abnormalities of gastric MALT lymphoma after successful eradication of Helicobacter pylori [66,67]. One large cohort with 108 patients with minimal histological residuals after eradication showed a favorable disease course in 94% of patients with low rates of progression (5%) and transformation (1 patient) during a follow-up time of 42.2 months [67].

Computed tomography (CT) of the chest and abdomen is the current method of choice to determine the dissemination of disease. Radiographic investigation of the salivary glands and orbitae is also recommended since multi organ involvement can be observed. For this, magnetic resonance imaging (MRI) is a sensitive approach; nevertheless, it is not definitively recommended by guidelines [6].

In contrast to DLBCL and Hodgkin lymphoma (HL), in which Fluorine-18 (18F) fluorodeoxyglucose (FDG) positron emission tomography (PET)/computed tomography (CT) is the imaging tool of choice for staging, restaging, and evaluation of treatment response [68], its role in EMZL without histologic transformation is not yet clear. One excessively debated point is the 18F-FDG avidity of EMZL, which can depend on several factors, including the resolution power of the used device and intrinsic characteristics of the tumor [69]. Nevertheless, technical improvements in equipment and growing research efforts have provided promising results. As an example, 18F-FDG-PET/CT can be very sensitive in detecting EMZL lesions in tissues with low homogeneous physiologic [18F]. FDG-uptake such as subcutaneous tissue and lungs. In addition, it shows good detection rates for salivary glands and thyroid manifestations [70]. However, its use in gastric EMZL remains investigational since physiological and/or inflammatory 18F-FDG-activity in the stomach can mask malignant lesions [69,71]. Current guidelines suggest its use in cases where only local therapy is intended [11,58].

As EMZL of the lung or intestinal tract can disseminate into the stomach, gastroscopy is recommended [11] in both patient groups as it seems to be exclusive for these [72].

Bone marrow involvement varies from 2 to 20%, and it is especially rare in gastric EMZL (2% of cases). Considering that, routine bone marrow biopsy is not currently recommended in guidelines; nevertheless, it may be of use in non-gastric EMZL when only local therapy is under consideration [11,72,73]. 

Similar to follicular lymphoma and HL, the choice of therapy for EMZL varies significantly according to the disease’s stage. As EMZL may persist as localized and asymptomatic for longer periods, precise staging is fundamental for follow-up, especially in cases where infectious agents have been eradicated—after local therapy and in cases in which a watch-and-wait strategy is proposed. Currently, there is no consensus regarding a preferable staging system for EMZL [11]. For gastrointestinal EMZL, there are currently three approaches: the Lugano and Paris staging systems and the Ann Arbor classification modified by Musshoff [74,75]. The Lugano system differs from the modified Ann Arbor classification through the inclusion of a stage describing penetration of serosa without lymph node involvement as the IIE stage. However, similarly to the modified Ann Arbor classification, stage II (both II1 and II2) in the Lugano staging system indicates lymph node infiltration [76]. Both classifications are mostly based on radiological findings [77]. The Paris staging system adapts the consolidated TNM classification and describes the lymphoma involvement in more detail with respect to depth of infiltration, nodal involvement and spread [74]. However, this staging system has not yet been confirmed in prospective trials. In the case of skin involvement, the preferred system is the TNM classification of cutaneous lymphoma other than mycosis fungoides and Sézary syndrome [78]. Table 1 summarizes the different staging systems used for gastric EMZL. The Ann Arbor classification continues to be the most used staging system in extra gastrointestinal sites [79].

The so-called MALT-international prognostic index (MALT-IPI) is a prognostic system developed by the International Extranodal Lymphoma Study Group (IELSG) for patients with EMZL [80]. Similarly to the International Prognostic Index, which is used in DLBCL [72], this system includes age (70 years or older), Ann Arbor stage (I and II vs. III or IV) and elevated lactate dehydrogenase (LDH) and stratifies patients into three groups: low, intermediate and high. Even though it correlates with event-free survival, its influence on treatment choice is still to be determined. In addition, patients with higher MALT-IPI are more likely to present with the early progression of disease within two years of diagnosis, which was correlated with poorer overall survival in the IELSG19 study [81]. 

## 2. Therapy

### 2.1. General Therapeutic Approaches

The therapeutic options for EMZL are based on three different general principles: watch-and-wait, local therapy and systemic therapy. However, as mentioned above, due to the important role of chronic inflammation and infection in the pathogenesis of EMZL, a fourth mainstay of management also deserves attention: eradication of causal agent [11,82].

In contrast to aggressive lymphomas, such as DLBCL, where immunochemotherapy is the mainstay of therapy [83], therapeutic options for EMZL may vary and depend on two important factors: stage of disease and primary site of involvement, which confers additional complexity to its management. For example, in gastric EMZL, *H. pylori* eradication is a fundamental aspect of the management and evidence-based recommendation in guidelines; nevertheless, its eradication is not recommended in extra gastric EMZL. In addition, surgery for this anatomic site is not recommended due to high associated morbidity; however, radiotherapy (RT) is an important option for patients with localized and/or residual disease after *H. pylori* eradication. Individual therapeutic options for different sites of involvement and stages will be discussed below.

Due to its indolent behavior, EMZL may remain localized for several years [6]. That explains why local therapy remains an important cornerstone of management, and it is the preferred treatment approach in earlier stages of disease in almost all involved sites.

### 2.2. Local Therapy

Radiotherapy is the preferred option for local therapy in almost all sites of involvement from EMZL. In gastric EMZL, involved-site radiation therapy (ISRT) has shown very good results with tolerable toxicity, and it is currently the preferred therapy for patients that do not respond to *H. pylori* eradication in several guidelines [11,84].

For other indications, reducing the radiotherapy dose to 24Gy has been shown to be non-inferior to standard doses [85]. Attempts of further reduction to ultra-low doses did not provide the same outcomes, [86]. One small study with 22 patients with ocular adnexal B-cell lymphoma (14 with EMZL) showed an ORR of 100% and a CR of 86% with an ultra-low dose [87]. Nevertheless, the long-term results of the FoRT trial showed higher relapse rates in patients treated with 4Gy. Therefore, 24Gy remains the internationally preferred dose [88]. The role of ultra-low-dose radiotherapy remains reserved for palliative cases [50]. 

As lymphomas are usually considered systemic diseases, the proposition of surgery as primary therapy seems paradoxical. Nevertheless, localized surgery offers curative potential, and in some anatomical sites, it may reduce disease-associated complications and comorbidity for patients with limited disease. Thyroid, lungs, skin, salivary glands, and intestines are sites where surgery alone might be suitable. In gastric EMZL, however, it is currently not recommended due to the high treatment-associated morbidity. 

### 2.3. Systemic Therapy

Even though systemic therapies are more often used in disseminated stages of diseases, current guidelines also discuss systemic therapy for patients with localized involvement [11,84]. In asymptomatic patients, a watch-and-wait regimen might be feasible until therapy is needed. The decision is, however, patient-oriented, and the exact moment when to initiate systemic therapy depends on several factors. In addition, since advanced stages cannot be cured, enrollment in clinical trials is strongly supported.

In general, more toxic regimens such as those containing anthracycline are not indicated in the first line since sufficient results may be reached with less toxic therapies. Nevertheless, not many agents have been systematically tested in EMZL. Therefore, many recommendations are based on results from small trials or extrapolation of data from other subgroups of the marginal zone or indolent B-cell lymphomas. 

Even though several regimens have been tested, mostly in phase II trials, and have proven efficacy in EMZL, there is still no consensus regarding the optimal first-line therapy for these patients.

### 2.4. Monotherapy Regimens

Continuous single-agent oral chemotherapy is known to be an option for gastric EMZL. Alkylating agents such as cyclophosphamide and chlorambucil have been proven to be efficient as monotherapy in several anatomical sites, with complete responses in around 75% of patients with gastric involvement after 12 months of treatment [89,90,91]. Of note, some studies have shown that the presence of t(11;18)(q21;q21) may predict poor response to oral alkylating therapies but not to rituximab or cladribine [49,51,52,53]. One study compared *H. pylori* eradication alone vs. *H. pylori* eradication plus chlorambucil in patients with gastric EMZL. After randomizing 110 patients, they found no differences in recurrence/progression rates after 5 years (11% for chlorambucil, and 21% for observation *p* = 0.15), underlining the importance of *H. pylori* eradication as the treatment of choice in gastric EMZL [92].

Rituximab has also been proven to be efficient in the treatment of EMZL [93,94]. One study with 35 patients showed an overall response rate (ORR) of 73%, which was significantly higher in patients who were chemotherapy naïve (87% vs. 45% *p* = 0.03) [93]. Purine analogs such as cladribine have also shown efficacy in EMZL, with response rates up to 100% and complete remission (CR) of 84% [95].

Lenalidomide has also been investigated in a phase II trial in patients with EMZL. Eighteen patients were enrolled, showing an ORR of 61% with a 6-month treatment schedule [96]. Moreover, late response onset was observed in one retrospective study with a median time to the best response for all responding patients (13 of 25; 53%) of 7.3 months [97]. Long-term follow-up studies showed sustained responses also in combination with rituximab [98]. Both studies have included a relatively large proportion of patients with localized disease. Since this later onset of response has been observed, it seems reasonable to investigate the effect of this drug in patients with lower stages—and without clinical urgency—especially those with contraindications for radiation.

Lenalidomide is an immunomodulatory drug (IMiD) with multiple mechanisms-of-action, meaning that it not only affects cancer cells but also stromal and immune effector cells [99]. Moreover, it has been shown that it inhibits angiogenesis, activates immune-effector cells and shifts cytokine production, all leading to an influence on the tumor microenvironment [99,100,101]. Its immunomodulatory effect, especially over immune-effector cells, is an important anti-cancer mechanism; nevertheless, as many patients with EMZL may present with concomitant autoimmune diseases, its use may lead to immune-mediated side effects or even disease flare. Of note, in one study of lenalidomide monotherapy, one patient with underlying Sjögren’s syndrome developed symptoms consistent with a tumor flare in a parotid MALT lymphoma during later stages of therapy [96]. Nevertheless, four patients had documented autoimmune diseases, which remained clinically unchanged during treatment with lenalidomide.

Clarithromycin has also been shown to be active in patients with MALT lymphoma [102,103,104]. One phase II study with 23 patients with EMZL without *H. pylori* and *C. psittaci* infections investigated the efficacy and safety profile of clarithromycin. Patients received oral clarithromycin 2 g/day, once daily, days 1–14, every 21 days. Most patients (17/23) had stage I disease, with six patients reaching a CR, and ORR was 53% with good tolerability. Nausea was the commonest side-effect, but it was manageable and did not require dose reduction and QT prolongation was not recorded [103].

### 2.5. Radioimmunotherapy

In cases where patients are not eligible to receive immunochemotherapy due to age or comorbidities, radioimmunotherapy might be an option as a chemotherapy-free approach. As an example, ^90^Y radioimmunoconjugate ibritumomab tiuxetan (^90^YIT) showed efficacy in patients with indolent B-cell lymphomas [105]. A phase II study showed an ORR of 87.5% 12 weeks post-therapy in 16 patients with previously untreated MZL with a 5-year OS of 71.8% [106]. YIT, which is a radio-conjugated murine monoclonal antibody, is FDA-approved for r/r FL or as consolidation in FL after first-line chemotherapy [107]. 

Moreover, iodine-131 (131I)-rituximab chimeric anti-CD20 antibody radioimmunotherapy achieved CR rates and a high ORR in patients with relapsed or refractory indolent NHL. In a multicenter phase II study by Leahy et al., an ORR of 76% was shown in 91 patients with r/r indolent NHL [108]. These studies show the efficacy of radioimmunotherapy in MZL as a treatment option in selected cases. Radioimmunotherapy, with its mild toxicity, might be an attractive single-course therapy in MZL patients of older age. However, an initial bone marrow biopsy is mandatory because of the potential of radioimmunotherapy to cause hematologic toxicity due to bone marrow irradiation.

### 2.6. Combination Therapies

Even though monotherapy is a feasible approach, combination therapy, especially as immunochemotherapy, is of particular interest due to the promise of improved outcomes with small additional toxicity. The IELSG-19 was a large randomized phase III trial in patients with EZML that investigated the combination of rituximab and chlorambucil compared to both compounds as monotherapy [91,109]. In total, 454 patients were included in the study, with a 1:1:1 randomization. Combination therapy led to a significant better event-free survival (EFS) after five years with 51% (95% CI, 42 to 60) for chlorambucil monotherapy, 50% (95% CI, 42 to 59) for rituximab monotherapy, and 68% (95% CI, 60 to 76) for the combination (*p* = 0.0009). Progression-free survival (PFS) was also significantly better with the combination (*p* = 0.0119). Both therapies were well tolerated. As expected, the combination arm had slightly higher hematologic toxicity events. In addition, one phase II study evaluated the efficacy of the combination chlorambucil and rituximab followed by rituximab maintenance every two months for two years with a CR rate of 80%, partial remission (PR) of 13% and 7% progressive disease by the end of treatment [72].

The therapy with rituximab and bendamustine (R-B) has shown efficacy in several indolent lymphomas [110], with one large phase III non-inferiority trial showing a preferable toxicity profile over R-CHOP (rituximab, cyclophosphamide, doxorubicin, vincristine and prednisone) and longer PFS [111]. However, patients with EMZL were not included in this study. To investigate the safety and efficacy of R-B in this entity, a single-arm phase II trial was performed, which included 60 patients with EMZL with gastric, non-gastric and multifocal involvement using a response-adapted strategy [112]. EFS at 2 years was 93% and at 4 years 88%, with no differences between involvement sites. Patients in CR after three cycles received another cycle, for a total of four cycles, and those with PR received three additional cycles, for a total of six cycles. Only 25% received six cycles [112]. Lymphopenia was the most frequently observed adverse event [112]. Further studies have confirmed these findings [113,114,115], also in pretreated patients [116]. Of note, one large study of an international consortium with 237 EMZL patients showed an ORR of 93.2% and a CR rate of 80%. Patients received a median of six cycles (range 1–8). Importantly, treatment with bendamustine is related to prolonged lymphopenia and risk of infections; *Herpes zoster* was frequently observed in patients treated with this regimen, and therefore, an adequate prophylaxis might be needed until immune reconstitution [114]. 

Of note, maintenance therapy with rituximab for two years following therapy with R-B demonstrated a statistically significant PFS improvement in comparison with observation in patients with MZL; nevertheless, there were no statistically significant differences in OS between both groups. However, this study did not include patients with EMZL [117].

The combination of rituximab and fludarabine (R-F) has also been investigated in patients with EMZL. One phase II study using a response-adapted strategy (4 × 6 cycles) showed ORR and CR at the end of treatment of 100% and 90%, respectively, with only mild toxicity [118]. On the other hand, a second phase II trial showed important toxicity, with only 58% of patients completing six cycles of therapy and four late toxic deaths due to infection, two related to bone marrow aplasia and two related to myelodysplastic syndrome [119]. ORR was 85% in this study. R-F with the addition of mitoxantrone has also been investigated, showing good ORR and prolonged responses (96.5% and 70.5% after 9 years, respectively) [120]. Nevertheless, the treatment-related toxicity seems to be higher than in other regimens [121,122,123]. 

In addition, one study compared R-B treatment with R-F in patients with relapsed indolent and mantle cell lymphoma, showing better results in the R-B arm. Of note, this study included only 18 patients with MZL, whose subgroups are not specified in the publication [124].

In order to reduce the toxicity of the standard CHOP protocol, the anthracycline-free R-CVP (rituximab, cyclophosphamide, vincristine and prednisone) regimen was investigated in retrospective and phase II studies both in gastric EMZL and advanced MZL (including EMZL) showing good ORR and tolerability [125,126]. Treatment with R-CVP followed by rituximab maintenance has also been tested in a phase II trial with patients with MZL, including EMZL, showing acceptable toxicity profiles with possible improvement of PFS (not a comparative study) of 81% at three years compared to 59% without maintenance [127].

Lenalidomide in combination with rituximab (R2) has also been investigated in patients with MZL. One phase II study including patients with follicular lymphoma (*n* = 50), marginal zone lymphoma (*n* = 30), and small lymphocytic lymphoma (*n* = 30) showed an ORR of 89% and 3-year PFS of 87% for patients with MZL [128]. Of special interest, patients with MZL showed, except for cough, unusual non-hematological AEs compared to patients with FL: cough/dyspnea/pulmonary (63%) and eye irritation (60%) as well as thyroid abnormalities 23%. The AGMT MALT-2 study evaluated the efficacy of R2 in 46 EMZL patients and found an ORR of 80% [129]. Herein, Table 2 shows an overview of immune- and chemotherapy regimens studied in EMZL. 

## 3. Specific Algorithms per Affected Site 

### 3.1. Gastric EMZL

Due to the high association of gastric EMZL with *H. pylori* (up to 90% of cases in previous years, but currently less frequent), its eradication is a fundamental step in the treatment of gastric EZML and is therefore still the first choice of therapy [11,58]. Eradication is effective in inducing long-term remission and may induce CR in up to two-thirds of treated patients [132,133]. In addition, there are also reports of successful antibiotic treatment in patients with *H. pylori*-negative gastric EZML. One possible explanation could be false-negative results in the search for *H. pylori* screening; nevertheless, as mentioned above, infection through *non*-*H. pylori Helicobacter* species could also be of importance [6,134]. The response rates in these patients to antibiotic eradication vary between cohorts [22,135,136]. One systemic review with a pooled analysis involving 110 patients showed a complete lymphoma regression in 15.5% of patients undergoing eradication therapy [136]. Considering the low-risk profile of eradication therapy compared to other therapy modalities and the growing understanding of *non-H.pylori Helicobacters*, eradication therapy in patients negative for *H. pylori* seems to be a reasonable approach.

Even though eradication is widely accepted, NCCN and ESMO guidelines differ slightly regarding the selection of which patient group should receive antibiotic treatment. In detail, NCCN considers *H. pylori* eradication as the first-line therapy of choice, mostly for patients with early-stage (I1, I2 or II1 Lugano) and *H. pylori*-positive disease and concomitant negativity or unknown status of t(11;18). In patients with *H. pylori*-positive disease and concomitant t(11;18), eradication should be accompanied by ISRT or rituximab treatment if ISRT is contraindicated. In the context of the ongoing COVID-19 pandemic, it is important to take into consideration that B-cell depleting therapies can cause poor antibody production after vaccination against SARS-CoV2 [137]. Moreover, it seems that patients with lymphoma and persistent COVID-19 infection who were treated with B-cell-depleting therapies within the previous 12 months have a nearly double risk of prolonged hospitalization and more than double the risk of death [138]. Patients with early-stage and *H. pylori*-negative disease should receive ISRT. According to the NCCN guidelines, patients with stage II2, IIE and IV do not necessarily have an indication for H. pylori eradication. An important rationale for this is the fact that patients with t(11;18), involvement of submucosa or lymph node are less likely to respond to eradication therapy [49,139]. 

In contrast, ESMO guidelines recommend the eradication a priori for all patients independently of stage, translocation status or *H. pylori* positivity [11]. Of note, a specific therapy should be considered earlier for those patients with higher risk of non-responsiveness if eradication does not show lymphoma regression or if patients present symptoms. Moreover, the ESMO guideline prefers ISRT as local treatment since surgery is accompanied by higher morbidity.

There are several possibilities for antibiotic eradication of *H. pylori,* and the choice should be made according to local resistance profiles [140]. Common regimens for first-line treatments are the triple combination of proton pump inhibitors (PPI) with clarithromycin and amoxicillin or metronidazole [74]. The success of the eradication should be tested 6–8 weeks after starting therapy and at least two weeks after stopping PPI using a urea breath test or testing for antigen in stool [11]. If there is still proof of infection, a second-line eradication therapy should be considered. 

Complete lymphoma responses may be observed already after three months after eradication; nevertheless, slower responses are not uncommon. Therefore, reevaluation before two months after treatment is only justifiable if the patients present with clinical worsening or symptoms. Recommended criteria and intervals for follow-up are discussed below. If patients show relevant responses, a systemic therapy does not improve outcome and is therefore not indicated [140]. If patients fail to respond but persist as asymptomatic, watch-and-wait is even a suitable approach. If symptoms are present, a local therapy is normally indicated. After local therapy, therapeutic success should be evaluated. If there is still lymphoma persistence, a systemic therapy is usually indicated.

Another difference between both guidelines is the staging-guided therapy. While ESMO indicates therapy separating stage IV from the others, NCCN includes both II2 and IIE in the Lugano classification as higher stages grouped with stage IV, leading these patients to receive specific interventions more upfront in comparison with the ESMO guidelines. Table 3 summarizes the essential differences between ESMO and NCCN.

Watch-and-wait is a suitable alternative for asymptomatic patients with advanced-stage disease. Systemic therapy is to be considered if the patient meets any of the indications listed in Table 4.

### 3.2. Non-Gastric EMZL

Intestinal: local therapy in early stages (I and II) with surgery or radiotherapy has curative potential [141]. After surgery, locoregional ISRT may still be useful if there are positive margins, when the risk of collateral damage is acceptable; nevertheless, its use is limited by the ability to target the area of question [58]. Even though *C. jejuni* has been associated with this site of involvement, there is no current indication for antibiotic therapy [11]. Stages III and IV might be only observed, and if patients present with symptoms or other treatment indications, therapy is recommended [140]. Regarding colorectal MALT, there are many reports that show that, even in this case, a cure with antibiotic therapy, including *H. pylori* eradication is possible, even in *H. pylori*-negative cases [142]. However, as the treatment for colorectal MALT lymphoma is not well established, in some cases, endoscopic submucosal dissection (ESD) [143] or endoscopic mucosal resection (EMR) might be a feasible approach to cure small localized rectal MALT lymphomas [144].

Lungs: Since primary lymphomas of the lungs are rare, diagnostic approaches are similar to those of lung carcinomas [145]. Usually, extranodal involvement according to Ann Arbor staging is classified as stage IV; hence, EMZL of the lung (bronchus-associated lymphoid tissue lymphoma, BALT) is usually staged as stage I. As it is an extraordinary slow-growing lymphoma, watchful waiting until pulmonary function is impaired is a feasible approach in this special site [65,146]. Radiotherapy is also possible if the diagnosis is already confirmed. One large retrospective analysis showed that patients receiving local therapy have improved PFS compared to those with systemic therapies [147]. Extended rituximab schedules as a single agent have shown positive results in patients with lung EMZL in case series and case reports [148,149].

Ocular adnexa: Radiotherapy is the therapy of choice for patients with endangered vision and the localized stage [11]. As mentioned above, the preferred dose for radiation is 24 Gy, following the results of the recently published FoRT trial [88]. One retrospective study analyzed efficacy outcomes of patients with primary ocular adnexa EMZ [57]. Of the 70 patients with Ann Arbor stage I disease receiving radiotherapy alone, 68 achieved a CR. Four patients showed a local relapse, eight showed an extra orbital relapse and two patients showed both simultaneously [57]. For patients with the advanced stage, watch-and-wait is suitable if they are asymptomatic [150]. Systemic therapy should only be initiated if symptoms are present [11]. In contrast to primary vitreoretinal B-cell lymphoma, which typically harbors mutations in *MYD88* and *CD79b* [151], fine needle aspiration in EMZL of the ocular adnexa is usually insufficient, and therefore, surgery is the chosen method for diagnosis if clinical suspicion is present [152]. Nevertheless, complete surgical excision may be difficult to perform if the lesion is not restrained to the conjunctiva or lacrimal gland due to the risk of complications and morbidity [152]. In addition, complete excision does not lead to improved survival [150]. One option for patients that are not at risk of acute lymphoma complication is the *C. psittaci* eradication with antibiotics. Upfront doxycycline at 200 mg per day for at least three weeks is a commonly used regimen [153]. Results from phase II trials have shown ORR of 65% and 5-year PFS of 68% [154,155]. Responses can also be obtained in patients with no evidence of *C. psittaci* in biopsy; nevertheless, response rates are higher amongst those with proof of infection [156]. In addition, intralesional rituximab has shown efficacy in a phase II study [157].

Skin: EMZL of the skin are staged according to the TNM classification system for primary cutaneous lymphomas other than mycosis fungoides and Sézary syndrome. The T classification for cutaneous lymphoma reflects the extent/distribution of primary cutaneous involvement consistent with the definition of “T classification” by the AJCC/UICC (ranging from T1–T3b) [158]. For stage T1a lesions, both radiotherapy or surgery are curative treatment options. Topical therapy has shown efficacy only in small series, and its use may be advantageous for symptom control [159]. ESMO guidelines suggest watch-and-wait for all patients with stages other than T1a if asymptomatic. Patients with T1b and T2a with symptoms can receive local therapy with surgery or radiotherapy. One retrospective analysis showed no significant differences between patients undergoing surgery or radiotherapy, even though surgery was associated with more local relapses [160]. NCCN guidelines consider radiotherapy as the preferred treatment option [161]. Moreover, local therapy with intralesional rituximab application has been investigated and may be an option in particular cases [162]. In the ESMO guidelines, patients with lesions T2b are encompassed together with T3, and therefore, systemic therapy may be offered earlier if patients are symptomatic [73]. Even though *B. burgdoferi* has been associated with skin EMZL, there is no indication for antibiotic treatment in these patients. Even though there are anecdotal reports of lymphoma regression after antibiotic treatment [60], results are still conflicting [163]. 

Salivary glands: radiotherapy has shown consistent results for patients with limited disease. One retrospective study found no differences in patients treated with radiotherapy, surgery or both [164]. Nevertheless, another retrospective study from Stanford with 30 patients with different head and neck indolent lymphomas showed statistically significant longer freedom from local progression in those patients who received RT compared to those who did not [165]. They recommend a dose of 30–30.6 Gray (Gy) in 1.5–1.8 Gy fractions delivered to the primary tumor site and regional nodes for early-stage to prevent tumor progression. In addition, one randomized trial with 39 patients with early-stage EMZL of the parotid glands showed no statistically significant differences between patients receiving RT alone or combined with adjuvant chemotherapy (CVP), with CR rates of 100% [166]. In patients with Sjögren syndrome and salivary gland EMZL, rituximab might be a valuable choice since it is efficient in both diseases [167]. 

Thyroid: patients with localized disease benefit from both surgery and radiotherapy. Moderate-dose RT achieved CR in 84 of 85 patients in one retrospective trial with patients with different EMZL [82]. Thyroidectomy has also shown good outcomes in patients with stage IE and together with radiation led to CR in 90% of patients [168,169]. Current guidelines recommend both surgery and radiotherapy for stage I. In patients with stage II, chemotherapy and radiation have been shown to be effective [168].

## 4. Follow-Up and Assessment of Therapeutical Success 

Considering that watch-and-wait is a feasible and common strategy in the management of EMZL, adequate follow-up is necessary. Current guidelines recommend clinical, laboratory and radiological monitoring every 6 months [11]. Those patients with non-gastric EMZL who have completed their therapies can be followed according to the recommendations for other lymphomas.

In patients with gastric EMZL, the response should be accessed using the groupe d’ Etude des Lymphomes de l’ Adulte (GELA) scoring system because of its reproducibility and high degree of inter-observer agreement [170]. The recommended interval for reassessment after antibiotic treatment and/or local therapy/upfront rituximab is 3–6 months [11,84]. If there is no residual disease, patients should be followed up with endoscopy and histology. NCCN guidelines recommend follow-up endoscopy every 3–6 months for 5 years and then yearly if the patients reach a CR, whereas European guidelines recommend follow-up endoscopy every 6 months for 2 years followed by every 12–18 months. Of note, prolonged follow-up is especially important considering that chronic inflammation may increase the risk of secondary stomach cancer [171].

## 5. Novel Agents and Future Perspectives

Considering the indolent nature of EMZL, the possibility of chemotherapy-free regimens raises special attention due to its reduced toxicity and lower risk of secondary malignancies. Especially in the era of targeted therapies, a number of novel agents have been investigated for the treatment of lymphomas.

### 5.1. Bruton Kinase Inhibitors (BTK)

Ibrutinib has been investigated in a variety of indolent NHL, demonstrating positive results [172,173,174]. In MZL, one open-label phase II study that included 63 previously treated patients (32 with EMZL) investigated the efficacy of ibrutinib 560 mg as monotherapy [175]. The ORR in this cohort was 48% with a median PFS of 14.2 months and a discontinuation rate of 17%. Regarding safety, grade 3 or more infections occurred in 19% of patients (the most common treatment-emergent serious AEs were pneumonia (8%) and bleeding). AEs occurred in 59%, including one major bleeding leading to death. Recently, the long-term follow-up of this study showed an ORR of 58% after a median follow-up of 33.1 months. The median duration of response was 27.6 months, and the median OS had not been reached, underlining the potential of BTKi in the treatment of MZL. Of note, ORR for nodal, extranodal and splenic MZL was 47%, 63% and 62%, respectively [176]. Of note, ORR for chemotherapy-naïve patients treated with prior single-agent rituximab is better than for patients with prior chemoimmunotherapy (81% vs. 51%), including a CR rate of 19% vs. 8%. Response deepened over time; ORR 48% at 1 year to 58% at 3 years with CR rates 5% at 1 years to 10% at 3 years. These results led to the FDA approval for the treatment of relapsed and refractory (R/R) MZL [177]. The irreversible BTK inhibitor zanubrutinib has also been tested in patients with MZL. The phase II trial MAGNOLIA enrolled 68 patients with relapsed/refractory MZL, of which 26 had EMZL [178]. The ORR was 74.2% with a CR rate of 25.8%; median PFS at 15 months was 82.5%, and four patients discontinued treatment due to AEs, none of which was treatment related [178]. This trial graded the FDA approval for R/R MZL in 2021 [179]. Acalabrutib is currently being investigated in patients with MZL in a series of ongoing clinical trials: NCT04646395 is a phase II study of the IELSG that aims to assess the efficacy of the combination of acalabrutinib and tafasitamab, an anti-CD19 monoclonal antibody, in patients with MZL. In addition, NCT02180711 aims to investigate acalabrutinib alone or as a combination therapy (with rituximab and/or lenalidomide) in indolent NHL (iNHL) (R/R FL and MZL).

### 5.2. Phosphatidylinositol 3-Kinase (PI3K) Inhibitors 

As the PI3K pathway is involved in the activation of NF-kB, which seems to play an important role in the lymphomagenesis of MZL, its inhibition has also been investigated in MZL [64]. Idelalisib, the first PI3K inhibitor, which targets PI3Kδ and PI3Kd, was tested in a phase II study that enrolled 142 patients with R/R indolent B-cell lymphomas. In total, 15 patients with MZL were included [180]. The individual response rates for these patient group was not available; nevertheless, a later-published analysis revealed an ORR of 47%. Nine patients had EMZL, of which four had a PR and one had a CR [181]. More recently, other PI3K inhibitors have been tested in the context of iNHL but are not yet approved for the treatment of MZL: duvelisib and copanlisib. The first inhibits PI3Kγ, along with PI3Kδ and other isoforms, whereas the latter inhibits PI3Kδ and PI3Kα preferentially [64]. Duvesilib showed an ORR of 47% in a phase II clinical trial with 129 patients with R/R indolent lymphomas. It included 18 patients with MZL that showed an ORR of 38.9% [182]. Copanlisib was also evaluated in 142 patients with relapsed/refractory indolent B-cell lymphomas, of which 23 (16%) had MZL. ORR was 59% in the general population and 70% in MZL patients.% [183]. Non-infectious pneumonitis was the most common drug-related adverse event leading to discontinuation (11 in total with 5 discontinuations), followed by lung infection and hyperglicemia [183]. One ongoing phase II clinical trial (NCT03474744) is currently investigating the combination of copanlisib and rituximab in the treatment of patients with MZL. Moreover, umbralisib, which inhibits PI3Kδ and CK1ε was investigated in a phase II trial in MZL. Sixty-nine patients with R/R MZL were included, of which 38 had EMZL. ORR was 49.3% with a CR rate of 15.9%, and the median DOR and PFS was not reached. ORR for EMZL was 45%. The most common grade ≥3 TEAEs leading to discontinuation were diarrhea (6, 2.9%) and ALT/AST increase (5, 2.4%) [184]. Based on the results of this trial, the FDA granted its accelerated approval for this indication [185]. 

### 5.3. B-Cell Lymphoma-2 (BCL2) Inhibitors

Venetoclax, a BCL2 inhibitor, as monotherapy, has only been investigated in MZL in one phase I trial in patients with NHL, which only included three patients with MZL [186]. The combination therapy with ibrutinib was more extensively investigated for this indication in a phase II study that included 12 patients, 7 of which had EMZL. Seven patients had R/R EMZL and five were treatment naïve. At week 16, ORR was 58% (7/12) evaluated with CT and 84% (10/12) evaluated with PET/CT. One patient discontinued venetoclax due to drug-induced hepatits [187].

### 5.4. CAR-T Cell Therapy 

Chimeric antigen receptor (CAR) T-cell therapy has shown promising results in aggressive lymphoma and acute lymphoblastic leukemia. Currently, several ongoing studies are investigating the efficacy of CAR-T products in indolent lymphomas too. Exemplary, ZUMA-5 [188], a phase II study of axicabtagene ciloleucel in patients with R/R iNHL recently published the results of 148 patients who received the product, including 24 patients with MZL. ORR was 92% among efficacy-evaluable patients with iNHL (*n* = 104), with a CR rate of 76%. In those with MZL (*n* = 20), the ORR was 85% (60% CR rate) and median PFS was 11.8 months, and the estimated 12-month OS rate was 92.9% after a median follow-up of 12.1 months. Serious adverse events were observed in 74 (50%) patients. Death occurred in four patients, with one treatment-related death [188].

The ongoing TRANSCEND FL study (NCT04245839) with lisocabtagene maraleucel, which includes patients with R/R MZL and follicular lymphoma treated, might help to define the role of CAR T-cells in MZL as the results of ZUMA-5 regarding MZL might have been a little bit disappointing, the ORR was 85% (60% CR rate) compared to the higher ORR in patients with FL with an ORR of 94% (80% CR rate), and there was a higher rate of grade ≥3 neurological toxicity in MZL compared to the FL cohort of the same study (15%). Moreover, the median axicabtagene ciloleucel-associated toxicity was higher in patients with MZL than in those with FL.

### 5.5. Checkpoint Inhibitors 

Even though therapy with checkpoint inhibitors might be promising for NHL, there is still no current evidence supporting its use. Several ongoing clinical trials are evaluating the efficacy of checkpoint inhibition alone (NCT03498612) or in combination for patients with NHL, including MZL (NCT02332980).

## 6. Conclusions

With a mostly indolent behavior, EMZL allows for different therapeutic modalities with a good 5-year OS across multiple locations. Local therapy remains the standard of care in the early stages showing good results. In gastric lymphoma *H. pylori*, eradication is possible in all stages, and it is the treatment of choice, especially in those cases with proof of infection. Hence, eradication (antibiotics) is still a feasible first approach even in *H.p.*-negative gastric MALT lymphoma. Watch-and-wait is suitable in many cases, especially in compliant patients, in whom restaging can be performed in regular intervals as there is a—even though small—risk of histological transformation into a more aggressive lymphoma and/or of loss of follow-up [14]. Even though there is no consensus regarding first-line therapy in more advanced stages, several agents in monotherapy or in combination have proven efficacy and a tolerable safety profile. Due to the usually good prognosis of the disease, choosing adequate therapy is still challenging since a wide variety of individual patient and disease aspects have to be taken into consideration. Nevertheless, even though we are in the era of personalized oncology, the role of molecular markers in EMZL is still small and needs to be further addressed. In addition, as most studies regarding systemic therapies are non-comparative and included inhomogeneous populations, more specific conclusions regarding the efficacy of these systemic therapies are limited. Therefore, there is still a need for comparative studies that might bring more light to the decision-making process. In addition, the role of newer imaging modalities such as FDG-PET/CT, which is well established in aggressive B-cell lymphoma, is yet to be better defined, especially as there might be differences regarding overall detection rates in involved sites.

As small molecules, immunotherapy and targeted therapy changed the treatment landscape for not only B-cell lymphoma but for oncology/hematology-oncology in general, and future studies should include these agents as single-agent or combined approaches especially in patients not eligible for local therapy. Moreover, as phase III trials provide the highest level of evidence, efforts should be undertaken to include more patients in these kinds of trials.

## Figures and Tables

**Table 1:** Different staging systems for gastric EMZL.

Anatomic Involvement	Ann Arbor	Lugano Staging	Paris Staging System
Mucosa	I1E	I	T1m N0 M0
Submucosa	I1E	I	T1sm N0 M0
Muscularis propria	I2E	I	T2 N0 M0
Serosa	I2E	I	T3 N0 M0
Penetration of serosa involving adjacent tissues	I2E	IIE	T4 N0-2 M0
Abdominal local lymph nodes	II1E	II1	T1-3 N1 M0
Abdominal distant lymph nodes	II2E	II2	T1-3 N2 M0
Extra abdominal lymph nodes	IIIE	IV	T1-4 N3 M0
Disseminated extranodal involvement or infra- and supradiaphragmatic lymph nodes	IV	IV	T1-4 N0-3 M1 T1-4 N0-3 M2 T1-4 N0-3 M0-2 BX T1-4 N0-3 M0-2 B0 T1-4 N0-3 M2 B1
Different shades of grey highlight the differences in the stage classification (I, II, III and IV) between Ann arbor and Lugano staging systems.			

**Table 2:** Summary of evidence in EMZL.

Author and Year	Substance	Study Type	*n*	Population	ORR or CR
Hammel 1995 [90]	Chlorambucil or Cyclophosphamide p.o.	Not defined	24	Symptomatic gastric EMZL multiple stages	CR 75%
Simon 2006 [89]	Chlorambucil p.o.	Retrospective	33	Ocular EMZL stage IE	CR 79%
Zucca 2017 [91]	Chlorambucil monotherapy	Open label randomized phase III	131	Multiple stages, gastric and extra gastric EMZL	ORR 85.5%
	Chlorambucil + rituximab		132		ORR 94.7%
	Rituximab monotherapy		138		ORR 78.3%
Conconi 2003 [93]	Rituximab monotherapy	Phase II	34	Multiple stages, gastric and extra gastric EMZL, previously treated and naive	ORR 73%
Jäger 2002 [95]	Cladribine	Phase II	25	Multiple stages, gastric and extra gastric EMZL	ORR 100% CR 84%
Kiesewetter 2013 [96]	Lenalidomide	Phase II	18	Histologically advanced stages gastric and extra gastric EMZL	ORR 61%
Kiesenwetter 2019 [98]	Lenalidomide alone or in combination	Real world data	50	Multiple stages, gastric and extra gastric EMZL	ORR 72% CR 48%
Rummel 2005 [110]	Rituximab plus Bendamustine	Phase II	63 (6 EMZL)	Low grade NHL	ORR 83% for MZL
Morigi 2020 [113]	Rituximab plus bendamustine	Retrospective	65 (28 EMZL)	Untreated MZL	ORR 89.3% for EMZL
Alderuccio 2022 [130]	Rituximab plus bendamustine	Mostly retrospective	237	Mostly advanced stage EMZL. Frontline therapy	ORR 93.2% CR 81%
Kiesewetter 2014 [116]	Rituximab plus bendamustine	Retrospective	14	Previously treated EMZL	ORR 92.8% CR 71%
Salar, 2009 [118]	Rituximab plus fludarabine	Phase II	22	Untreated EMZL, multiple stages, gastric and extra gastric	ORR 100% CR 90%
Brown, 2009 [119]	Rituximab plus fludarabine	Phase II	26 (8 EMZL)	Mostly previously untreated MZL	ORR 85% (only EMZL not available)
Zinzani 2012 [120]	Fludarabine, mitoxantrone, rituximab	Phase II	143 (49 EMZL)	Untreated all stages	ORR 96.5% MZL: ORR 95.5% and CR: 87.4%
Cencini 2018 [123]	Fludarabine, mitoxantrone, rituximab	Retrospective	13	Eradication refractory gastric EMZL	CR 100%
Rummel 2016 [124]	Rituximab plus bendamustine	Phase III non inferiority	114 (10 MZL)	Relapse indolent and mantle-cell lymphomas	General 1Y PFS: 0.76
	Rituximab plus fludarabine		105 (8 MZL)		General 1Y PFS: 0.48
Kang 2012 [125]	Rituximab, Cyclophosphamide, Vincristine and prednisolone	Phase II	40 (28 EMZL; 5 gastric)	First line therapy MZL, stage III and IV	General ORR 88% (Gastric EMZL 100% Non-gastric 87%)
Aguiar-Bujanda 2014 [126]	Rituximab, Cyclophosphamide, Vincristine and prednisolone	Retrospective	20	Gastric EMZL with or without previous eradication; multiple stages	ORR 100% CR 95%
Becnel, 2019 [131]	Lenalidomid plus rituximab	Phase II	30 (11 EMZL)	Untreated MZL stage III/IV	General: 93% EMZL: 88%
Kiesewetter 2017 [129]	Lenalidomid plus rituximab	Phase II	46	Treated and untreated, all stages EMZL	ORR 80%

ORR: overall response rate; CR: complete response; 1Y PFS: one-year progression-free survival.

**Table 3:** Differences between European and American guidelines for gastric EMZL.

Differences According to	NCCN	ESMO
*Eradication*		
	*H. Pylori* eradication frontline alone only for early-stage, negative t(11;18) and *H. pylori* positivity	Upfront *H. Pylori* eradication alone for all stages, irrespective of stage and *H. pylori* status
	Patients with early-stage and t(11;18) should receive eradication followed by ISRT or if contraindicated or not possible rituximab instead	Patients with t(11;18) should receive upfront *H. pylori* eradication. Earlier local/systemic treatment if lack of improvement or symptoms
	*H. pylori*-negative patients should receive ISRT.	Upfront *H. pylori* eradication indicated. Primary local therapy possible. Earlier local/systemic treatment if lack of improvement or symptoms
*Staging*		
	Stages II2 and IIE follow the algorithm of stage IV	Stages II2 and IIE follow the algorithm of more localized stages
*Follow-up:*		
*after antibiotics*	*H. pylori* evaluation after 3 months	*H. pylori* test after 6 weeks starting eradication and 2 weeks after PPI
	Endoscopic restaging 3 months after antibiotics	First endoscopic restaging 2–3 after months documentation of *H. pylori* eradication
*If negative for lymphoma*	Endoscopy after three months, if persistent negative, follow-up every 3–6 months for 5 years, then yearly	Endoscopy and biopsy every 6 months for 2 years, then every 12–18 months
*If residual lymphoma is asymptomatic*	Observe for 3 months or ISRT	Observe for 3–6 months

**Table 4:** Treatment indications for residual gastric EMZL.

NCCN	ESMO
Candidate for clinical trial Symptoms GI bleeding Threatened end-organ function Bulky disease Steady or rapid progression Patient preference	Symptoms Overt progression Deep invasion Bulky disease Impending organ damage Patient preference
